# Risk Stratification in Young Patients With Channelopathies

**Published:** 2010-06-05

**Authors:** N Sreeram, U Trieschmann, M Khalil, M Emmel

**Affiliations:** 1 Department of Paediatric Cardiology. University Hospital of Cologne, Germany; 2 Paediatric Cardiac Anaesthesia and Intensive Care Medicine, University Hospital of Cologne, Germany.

**Keywords:** Channelopathies, Risk Stratification

## Abstract

Identifying the young patient at risk of malignant arrhythmias and sudden cardiac death remains a challenge. It is increasingly recognised that sudden death, syncope and aborted cardiac arrest at a young age in patients with a structurally normal heart may be the result of various ion channel disorders - the channelopathies. The approach to risk stratification involves a combination of the clinical presentation, taken in conjunction with the family history, genetic testing, invasive electrophysiological studies or other provocative tests where appropriate and feasible. A logical approach to risk stratification in some of the commoner channelopathies seen in paediatric practice is presented.

## Introduction

The recognition that several of the common causes of sudden arrhythmic death in young patients are the result of mutations affecting ion channels at the level of the individual cell (and hence the generic disease description of channelopathies) has led to increasing interest in genotyping. For clinical practice, it is of interest to investigate to what extent genotyping is of relevance in the management of an individual patient suspected or known to have a channelopathy, and to compare the results of such genetic testing with the use of clinical markers of risk for sudden death. We will provide a brief overview of the commoner symptomatic channelopathies which present in young patients.

## The congenital long QT syndrome

Since its initial clinical description, our knowledge of the disease entity, the cellular mechanisms that underlie the various forms of the disease (the number of different genotypes associated with the long QT syndrome is currently 12) and the association between specific genotypes and the risk of sudden death has expanded exponentially. The relationship between genotype and phenotype (the clinical expression of the disease) has been well studied. Initial studies focused on the specific ECG characteristics of the three commonest genotypes (LQTS types 1-3) which account for approximately 70% of patients with the long QT syndrome. Particularly in adult patients, the QT morphology was shown to be quite distinctive for each of these genotypes, although there are substantial variations in repolarization abnormalities observed in the same subject and in members of the same family indicating both the dynamic nature of the disease and differences in gene penetrance [1-4]. Such relative specificity has also been observed for  triggers for cardiac events (syncope or sudden cardiac death) and the clinical course (age at onset of cardiac events and risk for aborted cardiac arrest or sudden cardiac death). In studies of genotype - phenotype correlation, it has been shown that arrhythmias were triggered primarily by exercise or emotional upset in LQTS1, by emotional upset and particularly sudden auditory stimuli in LQTS2, while arrhythmia occurred primarily at rest or during sleep in LQTS3 [5-7]. Swimming was found to be a particularly important trigger for ventricular arrhythmia in LQTS1 [6,8,9]. The genotype has also been demonstrated to be important in determining the incidence of cardiac events, from early studies arising from the international LQTS registry [10]. The risk of cardiac events has been shown to be significantly higher in LQTS 1 and LQTS2 when compared with LQTS3, with events occurring at a younger age. The cumulative mortality however was similar regardless of the genotype, as patients with LQTS3 had a higher percentage of potentially lethal events [10]. As will be seen, these early findings have to a large extent been reconfirmed by follow-up studies of the different age categories of patients in the LQTS registry.

### Genotyping to assess risk of sudden death

Knowing the specific mutation, or the location of the mutation has been shown to improve risk stratification. For LQTS 1, Shimizu et al demonstrated mutation site-specific differences in the risk of lethal arrhythmia in a Japanese population [11]. They showed that patients with mutations in the transmembrane domain of the KCNQ1 ion channel had more frequent cardiac events (syncope, aborted cardiac arrest or sudden cardiac death) than patients with C-terminal mutations. They also had a greater risk of the first cardiac event occurring at a younger age. Several baseline ECG parameters affecting cardiac repolarization (Q-Tend, Q-Tpeak, Tpeak-end intervals) were also significantly longer in subjects with transmembrane mutations, with exaggerated response of some of these measures (Q-Tend and Tpeak-end) to sympathetic stimulation. The differences in the dynamic response of ventricular repolarization to sympathetic stimulation between the 3 main genotypes (LQT1 to 3) had already been previously established by Noda et al, who showed that the QTc was maximally prolonged as the RR interval decreased in LQTS1 patients, moderately prolonged in LQTS2 patients, and least prolonged in LQTS3 patients in response to intravenous epinephrine [12]. They speculated, justifiably, that this difference in the dynamic response of ventricular repolarization to sympathetic stimulation may explain why the trigger for cardiac events differs between the genotypes. Moss et al demonstrated that patients with transmembrane mutations were at increased risk of cardiac events compared with those with C-terminus mutations. In addition, the biophysical function of the mutation was also important in determining the phenotype. Patients with a dominant negative effect of the mutation on ion channel function (>50% reduction in function) had a more severe phenotype compared with those exhibiting haploinsufficiency (< or = 50% reduction in IKs potassium channel current) [13].  These genetic risks were independent of traditional clinical risk factors such as the manifest QTc interval on the ECG, suggesting that variability in the electrophysiologic effects of the different mutations contributes to the variability in the risk of life-threatening cardiac events [14]. This in turn suggests that it is not sufficient to know the genotype, but knowledge of the specific mutation and its biophysical function is essential for risk stratification. For LQTS2, similar genotype-phenotype correlations have been established. It has been shown that subjects harbouring pore mutations have a more severe clinical course and a higher frequency of arrhythmic events occurring at a younger age when compared with those with nonpore mutations [15]. In a more recent study, missense mutations in the transmembrane pore (S5-loop-S6) region were associated with the highest risk of clinical arrhythmia [16]. Further studies involving the US, Japanese and Netherlands LQTS registries are currently underway to further analyse risk associated with specific mutations in the HERG gene.  Preliminary data for LQTS3 subjects with SCN5A sodium channel mutations also suggest that the location of the mutation and its biophysical function may be important determinants of the clinical phenotype [17,19], although further data from the combined LQTS registries are awaited.

Even more interestingly, and further supporting the case for detailed genetic studies as part of the diagnostic work-up in assessing risk, it has been shown that specific mutations are associated with unusual clinical severity. The KCNQ1-A341V mutation was shown in a founder population in South Africa to have a severe phenotype [20]. While the QTc (if >500 ms) also affected the phenotypic expression, when matched for QTc with LQT1 database patients, the presence of a KCNQ1- A341V mutation was still associated with a larger probability of experiencing a cardiac event. Additional studies on subjects from different ethnic backgrounds have confirmed the unusual clinical severity associated with the KCNQ1-A341V mutation, suggesting that mutation-specific behaviour exists independent of ethnic or genetic background. The KCNQ1-A341V mutation carriers were more likely to have cardiac events at a younger age, and also had a longer QTc [21].  Given the wide variations in QTc in individuals carrying the same mutation, it is also evident that additional genetic or environmental influences play a role in modifying both the QTc and the risk of sudden death. A lower resting heart rate has been shown to be protective, suggesting that individual autonomic make-up modulates phenotypic expression  [22,23]. Whether a blunting of the autonomic response in individual subjects (conferring a protective effect) may be affected by specific adrenergic gene receptor polymorphisms has been the subject of subsequent investigations [23]. The clinical phenotype is not completely explained by the electrophysiologic effects or biophysical properties of the mutation, which has been shown to have a dominant negative effect on IKs. In the absence of detailed information on possible modifier genes, identification of the specific mutation alone is therefore insufficient in predicting individual risk. Other modifier genes which affect the severity of clinical expression of LQTS have been subsequently identified. These may vary from coinheritance of two independent mutations, either of which when inherited alone have a mild phenotype but when present in combination produce severe clinical manifestations [24], to the association of LQTS mutations with certain single nucleotide polymorphisms, occurring with varying degrees of frequency in the general population but which in combination produce a severe clinical phenotype [25-27].

Specific single nucleotide polymorphisms, often occurring commonly in specific populations, have also been associated with a higher incidence of arrhythmic events and sudden death. The S1103Y polymorphism in the SCN5A gene is present in between 10 to 13% of healthy African Americans, and is the result of a single nucleotide substitution of a cytosine (c) for an alanine (a) in the second position of codon 1103, resulting in an amino acid change (serine for tyrosine in amino acid position 1103). It is associated with a markedly increased risk of arrhythmias in unrelated African American adults with arrhythmias [28], with QTc prolongation and syncope, and with an eightfold increase in the risk for sudden arrhythmic death in young African Americans [29]. It is also overrepresented in the sudden infant death syndrome, and in autopsy-negative sudden unexplained death in subjects older than 1 year in this specific population [30,31].

To summarise, the established yield of genetic testing in clinically irrefutable cases of LQTS is high [32]. There remains however, a considerable chance that a positive genetic test is a  false positive, and this is to some extent ethnicity dependent. In turn, this also means that as the clinical probability of LQTS decreases the probability that an identified mutation is non-causative correspondingly increases [33]. The nature of the mutation (nonsense, frameshift, splice-mutations or missense mutations) and the location of the mutation will all affect pathogenicity. The role of additional modifier genes in determining phenotypic expression in different individuals with an identical mutation also needs further elucidation.

### Clinical risk stratification in the LQTS

Several studies in the different age categories have been made possible by the establishment of various LQTS registries. This has in turn made possible risk assessment for SCD/ACA (sudden cardiac death/aborted cardiac arrest) events based on several clinical and ECG criteria. Some of these will now be briefly described.

The risk factors for a cardiac event during the first 12 months of life (SCD/ACA/syncope) include a QTc ≥ 500, a resting heart rate of ≤ 100 beats/minute, and female sex. The risk for a subsequent SCD/ACA remains high, as established during a 10 year follow-up of this subset of patients, and beta-blocker therapy is only partially protective [34]. This has also been borne out by observations in other studies with shorter duration of follow-up [35]. Bradyarrhythmia (sinus bradycardia or functional 2:1 AV block) are also common in this population [36]. Long QT syndrome patients who experience potentially lethal clinical events in the first year of life are at high risk for similar events in the first decade of life, and additional therapies such as permanent pacing, left cardiac sympathetic denervation or early implantation of a defibrillator need to be considered on an individual basis. There is also an association between long QT syndrome and sudden infant death syndrome (SIDS), and routine newborn ECG screening has been advocated to identify infants at risk [37]. A detailed description of the risks and benefits of such an approach are beyond the scope of this review, but may be found in the following reviews [38,39]. It is to be anticipated however that routine ECG screening of siblings and other close family members of the index patient presenting in infancy will be more routinely undertaken, resulting in early identification of potentially affected, but as yet asymptomatic, individuals. Whether early institution of beta blocker therapy in individuals thus identified will be protective, is at present unknown. Sudden death of a sibling (at any age) has been thought to be associated with a higher risk of death in the LQTS population. This has however not been confirmed in an LQTS registry study of first- and second-degree relatives of probands. Sibling death was not significantly associated with  increased risk of SCD/ACA; instead, the risk of adverse events in relatives was determined more by individual risk factors which included a QTc ≥530, a history of syncope, and gender [40]. QTc was highly predictive of ACA/SCD. A personal history of syncope, particularly if syncope had occurred within 2 years, was also strongly associated with ACA/ death. The effect of gender was time-dependent. The risk of ACA/death/any cardiac event was higher in boys than in girls at a young age, but this relationship changed from late adolescence onwards, when women had a higher risk than men [40]. To conclude, severe symptoms in a close relative cannot be used as an indicator of personal risk for other family members who may have the same genotype, although such subjects are more likely to be treated with beta blockers from a young age [40].

In children aged 1 to 12 years, boys were at a significantly increased risk of ACA/SCD. The risk factors for boys included a QTc>500 and prior syncope (with recent syncope within the previous 2 years carrying a higher risk) whereas prior syncope (recent syncope being more risky than remote syncope) was the only significant risk factor in girls [40]. Routine beta blocker therapy was clearly protective, and was associated with a significant reduction in the risk of life-threatening cardiac events in this age group. Regardless of genotype, a family history of SCD did not predict a higher risk of cardiac events in childhood. Similar considerations apply to the adolescent population (aged between 10 and 20 years). Syncope (both timing of syncope and number of syncopal events) was a significant risk factor for predicting ACA/SCD, with recent (within the last 2 years) syncope and higher number of syncopal events during this period carrying a higher risk [40]. A QTc≥530 was associated with increased risk. The effect of gender was age-dependent, with boys at higher risk in the age category 10-12 years, and no significant difference in gender-related risk being observed between 13 and 20 years risk [42]. The predominance of life-threatening events in boys during childhood and early adolescence may be the result of environmental (increased sport participation/ intensive physical activity), hormonal (opposing effects of estrogens and androgens on ventricular repolarization) or genetic (modifier genes not shared by boys and girls) influences risk [42]. Data on the specific genotype (LQTS1 vs LQTS2 vs LQTS3) did not contribute significantly to the outcome, as syncope was not used as one of the cardiac end-points unlike previous genotype based studies, but rather as a time-dependent covariate to assess the end points of ACA/SCD [10,42].

Beyond 40 years of age, women with a QTc ≥470 are at a higher risk of SCD/ACA, while in men event rates were similar in the various QTc categories unaffected (QTc<449), borderline (QTc 440 to 469) and electrocardiographically affected (QTc ≥470). Recent (<2 years in the past) syncope was the predominant risk factor in affected subjects, and those with a positive mutation had a significantly higher mortality, particularly those with an LQT3 mutation [44]. After the age of 60 years, the risk of death due to LQTS competes with other disease entities which may lead to death. Even in this older population, a trend towards lower mortality was observed in patients treated with beta blockers, although this may have been the result of multiple protective mechanisms. Timely ICD implantation should obviously be considered in high risk patients remaining symptomatic despite beta blocker treatment.

### The long QT syndrome in patients with other phenotypic anomalies

Patients with associated phenotypic manifestations (Jervell and Lange-Nielsen syndrome in its homozygous or compound heterozygous state and associated with sensorineural deafness, Timothy syndrome which manifests skeletal abnormalities, syndactyly, structural heart disease, autism and immune deficiency with predisposition to sepsis usually have more severe clinical forms of the congenital long QT syndrome. Especially with the J and L-N and Timothy syndromes, they are also less likely to respond to beta blocker therapy alone, and early defibrillator implantation appears to be mandated in this population [44,45]. The QTc (if ≥550) and a history of syncope during the first year of life appear to be predictive of the degree of risk in individual patients with the Jervell and Lange-Nielsen syndrome [44,46]. In contrast, the Anderson-Tawil  syndrome which may be associated with dysmorphic features, periodic paralysis and propensity for ventricular arrhythmias (PVCs and bidirectional ventricular tachycardia rather than torsade de pointes), has a generally more benign clinical course in terms of arrhythmic death [47]. The majority of these syndromes may be suspected from the presence of these characteristic clinical findings, and appropriate therapy decided upon.

### Do we need to know the genotype when selecting therapy?

The arguments against routine genotyping for risk stratification and for selecting therapy in patients with long QT syndrome can be made on several grounds [48]. As seen above, clinical risk stratification is quite effective, when one considers the clinical presentation (syncope/ACA versus no symptoms) in combination with the QTc (if >500) [49,50]. The demographics of LQTS have also changed remarkably, with increased awareness of the disease and consequent early diagnosis. Unlike the view held prior to genotyping and the establishment of genotype-phenotype correlations, LQTS is associated with a low rate of SCD/ACA, and the majority of patients are asymptomatic carriers. A significant proportion of phenotypically affected patients and their families cannot be identified at present by genetic screening alone, and given the varied clinical expression of symptoms (and QTc) in different family members carrying the same genetic mutation, therapy and clinical risk stratification cannot be standardised based on the identification of a specific mutation. Most importantly, there are only a limited number of therapies available which may prevent sudden arrhythmic death (beta blockers, ICD implantation, and probably selective left cardiac sympathetic denervation). Beta blockers have been shown to be highly effective (given appropriate patient compliance) for LQTS 1 and LQTS2, and for the subset of LQTS3 patients who have clinical events mediated by excessive adrenergic stimulation [51]. It seems reasonable therefore to recommend routine beta blocker therapy in all patients with the clinical diagnosis of LQTS, without knowledge of the genotype. The majority of LQTS3 patients (who form a small subset of the entire LQTS population) without adrenergic mediated events remain asymptomatic well into adult life, and perhaps do not require routine prophylactic therapy. The ICD may therefore be reserved for patients with persistent symptoms (recurrent syncope) despite beta blocker therapy, and those presenting with ACA. Data from children obtained in the era of ICD implantation tend to confirm these observations [50]. Early identification of the disease and appropriate beta blocker therapy in combination with necessary physical restrictions and avoidance of QT prolonging medications (comprehensive lists of such drugs may be found on several websites such as www.azcert.org and www.Torsades.org) may all play a role in reducing mortality. Efficacy of beta blockade may require additional investigations such as exercise testing or epinephrine challenge [53,54]. There are even data suggesting that the differential response to epinephrine provocation helps to distinguish between the three major LQTS genotypes, allowing the application of presumptive genotype-specific treatment strategies  [55]. While the current clinical practice tends to favour ICD implantation in young patients with a proven LQTS3 genotype, there is no evidence at present that this aggressive approach is warranted if the majority of LQTS3 patients remain asymptomatic well into their 40s.

## The Brugada syndrome in paediatric practice

Risk stratification in adults with the Brugada syndrome has been extensively investigated [56-60]. In contrast, risk stratification in children is hampered by several factors. The disease is rare in large segments of the world's population; it often does not manifest clinically in childhood, and in the absence of a positive family history, identification of the index case in childhood can be difficult. Genetic testing provides a positive result in only approximately 30% of patients with the clinical phenotype of Brugada syndrome. While the ajmaline (or flecainide) tests have been used to identify patients who might have Brugada syndrome, a positive test does not predict the risk of future clinical events, and routine testing in childhood in asymptomatic individuals has generally not found favour, except in instances where there is an adverse family history and a negative genetic study, and where the parents are anxious to know whether their offspring has the disease [61,62]. Finally, therapeutic options, even after confirmation of the clinical phenotype by provocative tests, are limited.

Data on Brugada syndrome in children, apart from small case series or single reports are predominantly limited to a single multicenter study [63,64]. Thirty children aged <16 years were identified from 13 participating centers. All of them had a type 1 ECG either at rest or following drug challenge, with 10/11 symptomatic patients having a spontaneous type 1 ECG. In contrast to adult data, there was no male preponderance in this population (again not unexpected, as one would expect the sex hormones to have an unimportant role in this predominantly pre-pubertal population), and supraventricular arrhythmias were quite common. Episodes of syncope or SCD were also commonly associated with fever, emphasising the importance of rapid antipyretic therapy in this population. It has been shown in several studies that there are temperature-dependent modifications in sodium channel properties, which may underly the propensity for atrial or ventricular arrhythmias; the reason why this is particularly so in children is as yet unclear  [65,66]. Brugada syndrome was also present in at least 1 family member in 25/30 (83%) of children (including a family history of sudden death in 10/25 children). However, a family history of sudden death did not predict an adverse outcome, with the majority of children with a family history of SCD being asymptomatic at the time of assessment. Being a highly selected population, there was, not unexpectedly, also a high incidence of SCN5A mutations. Apart from the association of potentially life-threatening events with fever, the study also suggested that quinidine might be effective in preventing potentially lethal arrhythmias in children, and might even be considered as a bridge to eventual ICD implantation.

### Risk stratification in children with Brugada syndrome

#### Provocative testing

While drug testing may help to establish the diagnosis of Brugada syndrome, there are no data to show that a positive drug test alone, using either ajmaline or flecainide correlates with symptoms or with risk of sudden death. Exercise testing plays no role in risk stratification, as the majority of syncopal or sudden death events occur at rest. The role of invasive EP study is similarly controversial. Based on meta-analyses of large adult studies, the role of routine EP testing for the purpose of risk stratification has generally been abandoned. It is however as yet unclear (apart from the possibility of a selection bias towards more severe cases in the series reported by the Brugada brothers) why there is such a discrepancy between their data and other studies [67-70]. There is general consensus that a negative EP study has a good negative predictive value, particularly in previously asymptomatic individuals [67]. The majority of asymptomatic individuals with a negative EP study (non-inducibility of ventricular arrhythmia) remain asymptomatic at follow-up. We have largely abandoned invasive EP studies in young children with a positive family history of Brugada syndrome, as these have invariably been negative. Our standard EP protocol for these patients has been to pace from 2 right ventricular sites, with upto 3 extrastimuli with a minimum coupling interval of 200ms; it may be argued that this protocol is not aggressive enough, and that stimulation of epicardial sites also needs to be considered. To date however, we have had no inducible VT using this protocol, and none of the children has died suddenly or had a documented ventricular arrhythmia at follow-up. At present, we are implanting loop recorders (ILRs) in patients with a positive family history (where a family member has either died suddenly or has an ICD) who present with unexplained syncope. Despite this approach, we have not identified any child with ventricular arrhythmias documented by the ILR, where syncope was the presenting complaint. This suggests that syncope is common in the young population, and is in the majority of children unrelated to potentially lethal tachyarrhythmias.

#### Genetic testing

The cardiac sodium channel is the main determinant of impulse formation and propagation in the heart, and loss of function SCN5A mutations, with consequent slowing of cardiac conduction velocities result in the Brugada syndrome [71]. Other rare mutations involving the genes encoding subunits of the L-type calcium channel may also result in this clinical phenotype [72]. Currently, more than 100 mutations in 7 different genes have been associated with the Brugada syndrome  [73]. Genotype-phenotype correlations in the Brugada syndrome have been less well investigated because only approximately 30% of patients with the clinical phenotype have a positive genotype, suggesting the possibility of genetic heterogeneity. It is recognised however that patients with established SCN5A mutations may have a higher incidence of resting ECG abnormalities and a larger increase in QRS duration following the administration of sodium channel blockers [74]. The PQ and QRS intervals in lead V2 were also more markedly prolonged with aging in the SCN5A mutation-positive group during follow-up [75]. Histological studies also showed significant apoptosis in the ventricular myocardium in patients with SCN5A mutations, suggesting further that abnormal sodium channel function may result in cellular damage, and an increased risk for arrhythmic events [76]. More recently, there has been preliminary evidence to suggest that the type of SCN5A mutation (missense mutations M -  in which a single amino acid is replaced by an aberrant one versus premature truncations - T mutations  - where the sodum channel protein is truncated because of the presence of a premature stop codon) and the degree of reduction in INa may have an effect on the phenotype [77]. The proportion of patients who experienced syncope (presumed to be arrhythmic in origin) was higher in those with a T mutation than those with an M mutation, as was the proportion of families in whom SCD had occurred in a first degree family member at a young age. Even among the M mutations, the clinical phenotype was more severe in patients with more severe INa reduction (M mutations with >90% peak INa reduction versus M mutations with ≤90% INa reduction).  Further evidence of the potential clinical importance of genotyping is provided by the knowledge that specific SCN5A mutations are associated with a high risk of sudden death in selected populations  [78]. Certain ethnic-specific gene polymorphisms may also affect the clinical expression of the disease, although it is at present unlikely that this information can be incorporated into clinical practice [79,80].

#### Clinical risk stratification

The presence of a spontaneous type 1 ECG (usually recorded in the context of symptoms such as syncope or aborted cardiac arrest in the individual, or when the patient presents for routine screening with a positive family history of Brugada syndrome) appears to be the only reliable marker for possible adverse arrhythmic events. As the ECG can vary on a daily basis, it is important therefore to undertake routine clinical follow-up, and obtain 12 lead ECG recordings every 6 months in asymptomatic individuals. Where a type 1 ECG occurs in combination with symptoms the treatment pathways become clearer; ACA=ICD; syncope=quinidine ± ILR ± ICD. What to do in the presence of a spontaneous type 1 ECG in an asymptomatic individual is controversial. Adult studies have shown that there is a near-equal distribution of a type 1 ECG between symptomatic and asymptomatic subjects [57,59]. Further developments in our understanding of the genetics of the disease may help in risk stratification. In selected patients with adverse family histories, prophylactic oral quinidine therapy may be considered [81,82]. It is also important in such patients to avoid drugs that have been reported to induce the type 1 ECG and/or fatal arrhythmias. A list of these, and current recommendations concerning their use, may be found at www.BrugadaDrugs.org [83]. A variety of additional ECG markers have been identified, which are associated with an increased risk of ventricular arrhythmias. These include daily fluctuations in the standard and signal averaged ECGs [84], prolonged QRS interval in V6 of ≥ 90ms and prolonged r-J interval in V2 of ≥ 90ms [85]), fragmented QRS complexes [86,87], late potentials on signal-averaged ECGs [88,89], spontaneous changes in ST segments on continuous or multiple ECG recordings  [90], and increased  Tpeak-Tend duration and Tpeak-Tend dispersion [91]. The clinical utility of these parameters in general practice remains to be established.

Data from adult patients in whom ICDs have been implanted confirm that there is a low incidence of arrhythmic events at follow-up [92]. There is also a significant risk of device-related complications (approximately 9% per year), and it has been reconfirmed that recurrent syncope can occur in this population also in the absence of arrhythmia. In contrast to previous studies which suggested a good negative predictive value for EP studies, ICD follow-up data also show that the EP study may be limited even in this predictive function. The ICD however is protective, and none of the patients with an ICD died during follow-up [92].

## Catecholaminergic polymorphic ventricular tachycardia (CPVT)

### Genetic aspects

Familial forms account for between 30 and 50% of  CPVT, with both autosomal dominant and recessive forms having been recognised (dominant ryanodine RyR2 receptor gene defect - CPVT1 - and recessive calsequestrin 2 - CPVT2 - gene defects) [93,94].  The RyR2-encoded ryanodine receptor gene, mutations of which cause CPVT type 1 (CPVT1) is a relatively large gene, with mutations having been identified in up to 45 of of the 105 translated exons. Despite this large gene size, up to 65% of CPVT1-positive mutations can be discovered by selective analysis of 16 exons, which makes a tiered approach to genetic diagnosis feasible  [95].

### Clinical presentation

The syndrome has its clinical onset at a young age, and is characterised by ventricular arrhythmias occurring during exercise, typically when the heart rate exceeds a threshold value of between 120 and 130/minute  [96]. The initial presentation may be syncope, epileptic seizures secondary to ventricular arrhythmia and cerebral hypoxemia (which are often misdiagnosed as primary epileptic seizures), or sudden death. The disease is associated with a high mortality, with 30 to 35% of patients with the clinical disease dying before the age of 30 years. There is also often a strong family history of syncope or sudden death, occurring in upto 30% of patients [96]. Akin to LQTS1, ventricular arrhythmias may also be triggered during swimming, and CPVT1 causing RyR2 mutations were identified in up to 90% of patients with swimming-triggered arrhythmic events who were lacking sufficient clinical evidence for the diagnosis of LQTS [97]. RyR2 gene mutations have been identified at post-mortem in young individuals dying suddenly and unexpectedly [98,99] even when death occurred at rest, and may also be causal in a small proportion of sudden infant death syndrome [100]. This emphasises the importance of obtaining a genetic diagnosis where possible even after death, so that surviving relatives may be appropriately investigated and treated [101].

### Risk stratification

The resting ECG is normal, and invasive EP studies have no role. Diagnosis, risk stratification and therapy are all guided by the clinical presentation in the individual, and by the family history (sudden unexplained death in a first degree relative, or established diagnosis in a family member). Therapy consists of avoidance of exercise and beta blocker therapy. The response to beta-blockers is variable, and additional measures are often necessary [102,103]. In patients with persistent symptoms additional therapeutic approaches include the addition of a calcium channel blocker [104], selective left sided cardiac sympathetic denervation [105,106] and/or implantation of an ICD [107,108].
